# Quality of life of Chinese urban community residents: a psychometric study of the mainland Chinese version of the WHOQOL-BREF

**DOI:** 10.1186/1471-2288-12-37

**Published:** 2012-03-27

**Authors:** Ping Xia, Ningxiu Li, Kit-Tai Hau, Chaojie Liu, Yubo Lu

**Affiliations:** 1 Department of Social Medicine, School of Public Health, Sichuan University, Chengdu 610041, China; 2 Educational Psychology Department, Faculty of Education, The Chinese University of Hong Kong, Shatin, NT, Hong Kong; 3 China Health Program, School of Public Health, La Trobe University, Bundoora, VIC 3086, Australia; 4 Guangdong Provincial Hospital of Traditional Chinese Medicine, Guangzhou 510120, China

## Abstract

**Background:**

The short version of the World Health Organization's Quality of Life Instrument (WHOQOL-BREF) is widely validated and popularly used in assessing the subjective quality of life (QOL) of patients and the general public. We examined its psychometric properties in a large sample of community residents in mainland China.

**Methods:**

The WHOQOL-BREF was administered to 1052 adult community residents in a major metropolitan city in southern China. The structural integrity of the 4-factor model in confirmatory factor analyses (CFA) and the relationship of QOL with demographic variables were examined. Validity was assessed using the known-group comparison (229 with vs. 823 without chronic illness), item-domain correlations, and CFA using the ML estimation in LISREL.

**Results:**

Internal consistency reliability of the whole instrument (26 items) was 0.89, and the psychological, social, and environment domains had acceptable reliability (alpha = 0.76, 0.72, 0.78 respectively), while that of the physical domain was slightly lower (α = 0.67). The respective mean scores of these domains were 13.69, 14.11, 12.33 and 14.56. Item-domain correlations were much higher for corresponding domains than for non-corresponding domains, indicating good convergent validity. CFA provided a marginally acceptable fit to the a priori four-factor model when two matching content item pairs were allowed to be correlated; χ^2 ^(244) = 1836, RMSEA = 0.088, NNFI = 0.898, CFI = 0.909. This factorial structure was shown to be equivalent between the participants with and without chronic illness. The differences in means between these two groups were significant but small in some domains; effect size = 0.55, 0.15, 0.18 in the physical, psychological, and social relationship domains respectively. Furthermore, males had significantly higher QOL scores than females in the psychological domain, while individuals with a younger age, higher income, and higher education levels also had significantly higher QOL. Compared with the international data, the Chinese in this study had relatively low QOL scores with about 5% of males and 16% of females being at risk for poor QOL.

**Conclusions:**

This study has provided psychometric properties of the WHOQOL-BREF as used in China and should definitely be useful for researchers who would like to use or further refine the instrument.

## Background

The World Health Organization Quality of Life (WHOQOL) assessment group has defined quality of life (QOL) as the perception of individuals regarding their life position in the context of the cultural and value systems in which they live. QOL is related to their life goals, expectations, standards, and concerns [1,2]. Recent focus on QOL has resulted in a proliferation of assessment instruments and the development of relevant theories [3-5]. The WHOQOL-100 and its abbreviated version WHOQOL-BREF have been developed under the leadership of WHO over the past three decades [6-8]. In particular, the WHOQOL-BREF is relatively brief (26 items), convenient to use, and valid across cultures. Thus, it has been widely used in large-scale epidemiological surveys [9]. China joined the WHOQOL Group in 1995 and the team spent four years developing the Chinese version of these instruments. The Chinese WHOQOL-100 and WHOQOL-BREF were made available for researchers to use starting in 2000 [10-16].

Despite the substantial body of QOL studies being conducted around the world, the use of WHOQOL-BREF instrument in large Chinese samples is definitely insufficient. Following the analyses as conducted by Shek [17], a computer search (September, 2011) with the term "quality of life" showed 32331, 146729, and 15229 citations in PsycINFO, MEDLINE, and CINAHL respectively. These figures drastically reduced to 721 (721/32331 = 2.2%), 1271 (0.9%), and 314 (2.1%) respectively when we further limited the search to articles containing the term "Chinese". Among these, articles that contained both the terms "quality of life" + "Chinese" and used the WHOQOL-BREF were only 27 (27/721 = 3.7%), 40 (3.1%), 22 (7.0%) respectively. The present study was an attempt to fill in this gap of research using a relatively large sample of community residents in China. Although the basic psychometric properties of the QOL instruments were examined during the development of the version for the People's Republic of China [16], no subsequent large-scale systematic survey and analysis of the psychometric properties and factorial structure of the instrument has been conducted subsequently.

Chinese people have many unique characteristics that will probably challenge the utility of QOL instruments. As Shek [17] has pointed out, the strong Chinese emphasis on forbearance, endurance, humility, and interpersonal harmony, which stem from Confucianism and other cultural influences may lead to a greater tolerance of pain and adverse physical conditions than those of other cultures. Therefore, it may result in an artificial inflation of QOL measures--people appear to be more satisfied from their responses in the questionnaire than they are in real life.

Of particular interest is the question of how recent, rapid economic growth and massive rural-to-urban migration has affected QOL in China's population. The World Bank estimated that the China's gross domestic product (GDP) in 2010 was US$5.87 trillion, which put her the second in the world only after The United States [18]. Chinese cities have also seen a population surge as the proportion of people living in urban areas increased from 20% in 1981 to 44% in 2009 [18]. Taken together, these factors have fundamentally affected Chinese society and all aspects of living, and subsequently people's subjective perception of QOL.

Previous Chinese studies of QOL focused mainly on patients or particular populations such as AIDS patients, HIV-infected heroin users, Bell's palsy patients, workers exposed to coal dust, rural-to-urban migrants, and earthquake victims [10-15]. Thus, these studies provided little information on health-related QOL with reference to a standardized instrument (e.g., WHOQOL-BREF) for the general urban population in mainland China, and the psychometric properties of the WHOQOL-BREF in this population remain largely elusive.

One advantage of using a large sample of residents in this research is that we can use its score distribution to set a cut-off value (minimal standard), below which will be classified as low QOL. This will enable us to estimate the prevalence of people with low QOL in the population. Two commonly used cut-off standards for low QOL have been proposed, namely, "70% of the maximum score" and "one SD below the mean" [19,20]. Thus, according to these standards, a person with a score below 70% of the maximum score or more than one SD below the mean will be considered as having low QOL.

The objectives of the current study were, thus: (i) using a large sample of urban residents from southern China, to examine the psychometric characteristics of the mainland Chinese version of the WHOQOL-BREF by analyzing the floor/ceiling effect, its internal consistency, its convergent/discriminant validity, the factorial structure in confirmatory factor analysis (CFA), and its known-groups validity; (ii) to assess the relations between various domains of QOL and demographic background, and (iii) to estimate the prevalence of low QOL Chinese in this particular community sample.

## Methods

### Participants and procedures

This survey was conducted in Guangzhou which has a population of more than 12.8 million [21]. It is the capital city of Guangdong Province and is a major metropolitan area in Southern China with the third highest GDP among all cities in the country [21]. We chose districts with mixed population in terms of socioeconomic status and demographic characteristics which best represented the overall population of Guangzhou. Stratified cluster sampling was used to select six resident communities and recruitment flyers were distributed to the residents through the local community health workers. A total of 1090 urban residents aged 18 years or older were recruited in the year 2007, among which 1052 completed the questionnaires.

The questionnaire was administered through face-to-face household interviews by trained research assistants who were medical students proficient in both Mandarin (the official language) and Cantonese (the local dialect). Except for the illiterate participants (4.8%) who had the items read out to them, most participants completed the questionnaire at their own pace. Verbal consent was obtained from all participants and the research had been approved by the research ethics committee of the Guangdong Provincial Hospital of Traditional Chinese Medicine.

### Instrument

The WHOQOL-BREF was chosen for the present study for a number of reasons. First, it is one of the most commonly used generic QOL questionnaire simultaneously developed in diverse cultures, thus overcoming the typical problems relying on either the emic or the etic approach [1,22]. Second, due to its relatively short length, it is a very convenient instrument for large-scale surveys or clinical trials. Third, the official Chinese version for the PRC was developed and formally approved by the WHOQOL Group in 1999 and became available for use in mainland China at the planning stage of the present research. As described in a report from the WHOQOL Group, it is available in approximately 40 different languages for both developed and developing countries [8,23]. While other Chinese versions (including ones for Hong Kong and Taiwan) have also been developed and examined [24-27], the psychometric properties of the mainland China instrument remain largely unknown.

The mainland Chinese version of the WHOQOL-BREF [16] consisted of 28 items, including 26 standard items from the original WHOQOL-BREF and two additional items that were unique to the Chinese instrument. The 26 original items included two items on overall QOL and general health (the general facet on health and overall QOL). The remaining 24 items, on a five-point scale, could be classified into four domains: physical (7 items), psychological (6 items), social relationships (3 items), and environment (8 items) [6,8,9]. The two items specific to Chinese were: "Does family friction affect your life?" and "How is your appetite?", which were put at the end of the instrument [16]. To the team developing the Chinese WHOQOL-BREF, these items were believed to capture the important role of family and appetite as potential indicators of QOL in the Chinese culture. As recommended by the original Chinese developers, these two culturally specific items were analysed separately and not included in any domain score in order to maintain the comparability of the Chinese instrument with the standard WHOQOL-BREF [16]. The total score for each domain was converted to a score that ranged either from 4 to 20 or from 0 to 100, with low scores indicating poor QOL [9]. A domain was treated as missing when over 20% of its items were missing.

### Statistical analyses

Analyses of the frequency distribution at the item level were performed to assess the ceiling and floor effects. The internal consistency reliability of the instrument was measured by Cronbach's α, and the convergent and divergent validity of items were assessed by item-domain correlation coefficients. Discriminant validity was determined by *t*-tests which compared scores for various dimensions of QOL between community residents with and without chronic illness.

Confirmatory factor analysis (CFA) using the commercially available software LISREL (version 8.8) with the maximum likelihood estimation was used to examine the factorial construct validity of the instrument, whereas multiple-group analyses were used to compare the loadings, factor covariance, item uniqueness, and factorial means between the groups with and without chronic illness. We fitted the covariance matrix of the data (24 items) with the a priori four-factor model in CFA. Historically, the goodness of fit of the data to the model was assessed with the chi-square value derived from the minimum fitting function. This has been replaced by other more commonly used indices (e.g., RMSEA, CFI, NNFI) [28-31] with recommended cut-off values (e.g., RMSEA ≤ 0.08, CFI ≥ 0.90, NNFI ≥ 0.90) [28-31]. These cut-off values, however, should be taken as reference rather than as golden rules [31-34] because they are affected by the structure and complexity of the model, the number of indicators, etc. in the specific models being studied.

The relationships between demographic background and QOL were examined using both univariate (*t*-tests, one-way analysis of variance) and multivariate (multiple regression) analyses with the commercial software SPSS 17.0.

## Results

### Sample characteristics

Of the 1090 recruited participants, 1052 (96%; N_male _= 455, N_female _= 597) completed the questionnaire. The respondents comprised 823 participants (78.2%) without chronic illness and 229 (21.8%) with chronic illness. For this study, "chronic illness" was operationally defined as a medical condition diagnosed by a doctor at least six months before the study, for which either the symptom(s) still persisted or relevant medical treatment continued. In terms of age distribution, there were 667 (63.4%) in the younger group (age < 45 years), 193 (18.3%) in the middle age group (age 45-59 years), and 192 (18.3%) in the older age group (age > 60 years), with an overage mean age of 40.44 years (SD = 18, median = 34). Among the participants, 601 (57.1%) were employed, 125 (11.9%) were students, 236 (22.4%) were retired, and 90 (8.6%) were either casual workers or unemployed.

There were no significant differences in gender, family size, or monthly family income between participants with and without chronic illness. However, age (*χ*^2 ^= 3.3, *p *< 0.001), marital status (*χ*^2 ^= 65.9, *p *< 0.001), education level (*χ*^2 ^= 76.9, *p *< 0.001), and employment status (*χ*^2 ^= 137.9, *p *< 0.001) were significantly associated with chronic illness. Specifically, those with chronic illness were older, less educated, and more likely to be divorced and widowed. The participants revealed 35 identifiable physical and mental health conditions according to the International Classification of Diseases (ICD)-10 categories. Of the 229 participants with chronic diseases, 198 (86.46%) had one, 22 (9.61%) had two, and 9 (3.93%) had three or more chronic illnesses. The most prevalent conditions were hypertension (7.89%), diabetes (2.57%), chronic rhinitis (2.57%), and chronic gastroenteritis (2.19%). With regard to monthly family income, 22.0% of the participants received less than CNY(Chinese Yuan) 1500 (broadly described as low income; US$1 ≈ CNY6.50), 53.1% received CNY 1500-4000 (middle income), and 24.9% received more than CNY 4000 (high income).

### Data quality

The responses to each item of the questionnaire were fairly distributed across the full range of the scale, with no evidence of ceiling or floor effects for any item in the total data set (Table 1). Missing data was reasonably rare, with a total of only 9.2% of the 1052 participants reporting missing data for one or more of the items. The item with the highest rate of missing values (8.8%) was Item 21 "How satisfied are you with your sexual life?" All other items had a rate of missing values that was well below 0.3%.

**Table 1:** Distribution of responses (%) in the mainland Chinese WHOQOL-BREF (N = 1052)


**Items**		**1**	**2**	**3**	**4**	**5**
		**Very poor**	**Poor**	**Average**	**Good**	**Very good**

Q1	General QOL	1.2	5.1	54.6	34.3	4.8
Q2	General health	1.4	14.3	43.7	34.5	6.1
D1: Physical						
Q3	Pain and discomfort	1.5	17.3	24.5	45.7	10.9
Q4	Dependence medication	1.1	8.9	16.0	25.2	48.8
Q10	Energy and fatigue	2.1	15.2	35.4	37.3	10.1
Q15	Mobility	0.3	2.7	25.1	40.8	31.2
Q16	Sleep and rest	2.8	12.1	39.4	38.4	7.3
Q17	Activities of daily living	0.3	3.4	39.4	49.7	7.2
Q18	Working capacity	0.3	4.7	41.1	47.0	6.9
D2: Psychological						
Q5	Positive feelings	1.8	11.2	36.2	42.7	8.1
Q6	Spirituality, religion and personal beliefs	2.0	9.3	33.9	46.2	8.6
Q7	Thinking, learning, memory and concentration	2.0	13.9	44.4	32.9	6.7
Q11	Body image	2.4	15.2	52.5	23.2	9.4
Q19	Self-esteem	0.5	5.3	38.6	47.1	8.4
Q26	Negative feelings	0.6	6.9	45.9	36.8	9.8
D3: Social relationships						
Q20	Personal relations	0.7	3.2	36.6	51.9	7.6
Q21	Sex	1.0	4.0	50.0	36.3	5.8
Q22	Practical social support	0.4	4.2	43.6	46.5	5.3
D4: Environment						
Q8	Safety	3.2	13.5	43.3	35.2	4.8
Q9	Home environment	4.8	14.4	47.6	28.8	4.5
Q12	Financial resources	15.8	26.4	37.9	16.4	3.4
Q13	Information	6.4	27.9	38.2	24.3	3.2
Q14	Recreation and leisure	8.3	23.0	30.3	30.9	7.5
Q23	Physical environment	2.3	12.8	42.7	37.6	4.6
Q24	Access to health care	3.6	14.6	44.9	33.1	3.8
Q25	Transport	4.5	15.8	42.9	32.4	4.5
Q27*	Family friction	8.7	35.4	35.3	15.5	5.1
Q28*	Appetite	0.7	3.1	40.8	43.5	11.9

* Items in the mainland Chinese version only

### Internal consistency

As a measure of the internal consistency of the scale, Cronbach's α was reported for the total subject population and each sub-group (Table 2). For the total population, the Cronbach's α was 0.89 for the 26 items adapted from the original standard WHOQOL-BREF instrument and 0.88 for the 28 items that included the two additional questions for mainland Chinese participants. For the original 26-item (> 0.70) instrument, the Cronbach's α was acceptable for the psychological (0.76), social (0.72), and environmental (0.78) domains, but was only marginally acceptable for the physical domain (0.67). For this domain, the Cronbach's α would have increased to 0.71 if Item 3 ('pain and discomfort') and Item 4 ('dependence on medication') had been deleted. The Cronbach's α coefficient for people without chronic illness were much higher than for those with chronic illness (Table 2).

**Table 2:** Internal consistency reliability (Cronbach's α) of each dimension for different samples


**Sample**	**Physical**	**Psychological**	**Social relationship**	**Environmental**

Guangzhou (N = 1052)	0.67(0.71)^a^	0.77	0.72(0.45)^a^	0.78
Without chronic illness (N = 823)	0.66(0.70)^a^	0.77	0.75(0.47)^a^	0.79
With chronic illness (N = 229)	0.61(0.65)^a^	0.72	0.53(0.34)^a^	0.75
Taiwan Chinese (N = 1017)	0.76	0.70	0.68(0.72)^b^	0.75(0.77)^b^
Hong Kong Chinese (N = 848)^c^	0.76	0.80	0.67	0.78
Normative Sample (23 countries) (N = 11830)^d^	0.82	0.81	0.68	0.80

^a^In brackets: including two additional items for the mainland Chinese version

^b^In brackets: including additional items for the Taiwanese version: "Do you feel respected by others?" (social domain) and "Are you usually able to get the things you like to eat?" (environmental domain [25])

^c^Hong Kong data [24]

^d^Normative Sample (23 countries) data [9]

To analyze the two additional items specific to the Chinese instrument, we added them to the relevant domains based on their linguistic and semantic meanings after discussion with the experts who developed the Chinese WHOQOL questionnaires. Item 27 ('Does family friction affect your life?') was included in the social relationship domain, and Item 28 ('How is your appetite?') was added to the physical domain. Cronbach's α showed an increase in the 8-item physical domain that included Item 28, but dropped substantially below 0.70 in the 4-item social domain that included Item 27 (Table 2). Table 2 summarizes our findings in comparison with those from Taiwan [25], Hong Kong [24], and the original normative sample (23 countries) [9]. In general, our results were comparable with these studies on other Chinese populations.

### Convergent and discriminant validity

In itemized psychometric analyses, to support the convergent and discriminant validity of the items in the instrument, each item should have much higher correlations with items in its own domain and lower correlations with items in the other non-corresponding domains (Table 3). As can be seen from Table 3 (item convergence), 20 of the 24 (83%) of the items correlated at least 0.40 with its domain score and thus met the criterion for item convergence. Among the four domains, the items in the physical domain performed the worst and were in line with its lower Cronbach's alpha value. This might reflect the more heterogeneous nature of the items in the domain.

**Table 3:** Convergent validity of WHOQOL-BREF


	**Dimension**			
	
	**Physical**	**Psychological**	**Social relationship**	**Environmental**

Item Convergence^a^	4/7	5/6	3/3	8/8
Item Discriminant/Convergent Validity^b^				
(i) Intrascale item-corrected total correlations				
mean correlations	0.39	0.50	0.53	0.48
median correlations	0.41	0.45	0.53	0.50
Range	0.44	0.40	0.01	0.12
(ii) Interscale item-total correlations				
mean correlations	0.29	0.37	0.40	0.31
median correlations	0.34	0.36	0.40	0.30
Range	0.61	0.35	0.15	0.36
Correlations among Domains^c^				
Physical Domain	1			
Psychological Domain	0.53(0.57,0.70,0.71)	1		
Social relationship Domain	0.47(0.55,0.61,0.53)	0.52(0.60,0.73,0.73)	1	
Environmental Domain	0.40(0.54,0.72,0.57)	0.58(0.59,0.76,0.72)	0.50(0.61,0.71,0.65)	1

^a^The ratio of items to corrected-total correlated (i.e., item to corrected-total correlation) > 0.40

^b^For example, for the Physical Domain, 0.39 is the mean (whereas 0.41 is the median) of the 7 item-corrected total correlations for the 7 items within the Physical Domain; whereas 0.29 is the mean (0.34 is the median) of the 21 (7 × 3) correlations between the 7 items in the Physical Domain and the three other Domain scores

^c^in brackets: Chinese data [35-37]

Items correlated more strongly with items in the same (i.e., corresponding) domains than with those in other (i.e., non-corresponding) domains. Thus, for example, items in the physical domain correlated more robustly with the other items in the physical domain (average item corrected-total correlation for own domain = 0.39) than with those in the other three non-corresponding domains (average correlation = 0.29). Similarly, item-domain correlations for the psychological, social relationship, and environmental domains within the same domain were much higher than those for non-corresponding domains (Table 3). In general, the above results supported the a priori four-domain structure.

The Pearson correlation coefficients between the domain scores were high, ranging from 0.40 (physical and environmental) to 0.58 (psychological and environmental). The correlations among the various domains of the WHOQOL-BREF for different Chinese populations are summarized in Table 3[35-37].

A known group comparison method was adopted to provide further support for the discriminant validity of the instrument. Specifically, the QOL was compared between participants with (n = 229) and without (n = 823) chronic illness (Table 4). The results showed that the Chinese version of WHOQOL-BREF could discriminate people with and without chronic illness. The participants with chronic physical disorders showed significantly lower scores in the physical, psychological, and social relationship domains (all *p *< 0.05), but not in the environmental domain, as compared with the participants without chronic illness (Table 4). The effect sizes of the comparisons between the participants with and without chronic diseases were 0.55 for physical, 0.15 for psychological, 0.18 for social relationship, -0.04 for the environmental domains, and 0.40 for the general facet on health and overall QOL.

**Table 4:** Discriminant validity of WHOQOL-BREF(score range 0-100)


**Relation with chronic illness**	**Physical**	**Psychological**	**Social relationship**	**Environmental**

With chronic illness (Mean ± SD)	60.73 ± 11.94	58.91 ± 13.15	61.13 ± 11.79	52.54 ± 13.83
Without chronic illness (Mean ± SD)	67.46 ± 12.34	61.00 ± 14.15	63.78 ± 14.41	51.90 ± 14.72
t-values	7.36	2.01	2.85	-0.59
*p*-values	0.00	0.04	0.01	0.06
effect size^a^	0.55(0.15)	0.15(0.17)	0.18(0.16)	-0.04(0.17)

^a^in brackets: Taiwan data [35]

### Factorial construct validity

CFA with the commercially available software LISREL (version 8.8) was used to confirm the factorial structure of the WHOQOL-BREF items. When the fit of the data to the a priori model is acceptable, the proposed factor model is said to be applicable to the collected data. For items belonging to different factors in a factorial model, sometimes it is logical and necessary to allow their uniqueness to be freely correlated [38].

Results showed that the fit of the data to the four-factor model was only marginally acceptable (Table 5, II) and would be improved substantially if two pairs of items with matching content had been allowed to be correlated freely (Table 5, III); Δ*χ*^2^(2) = 565, ΔRMSEA = 0.013, ΔNNFI = 0.032, ΔCFI = 0.029. The two pairs of items were Item 5 ('enjoy life?') and Item 6 ('meaningful life') as well as Item 8 ('safe in daily life?') and Item 9 ('healthy physical environment?') The goodness-of-fit statistics with and without the correlated uniqueness were comparable to those reported in the original manual that described the construction of the instruments for various countries (Table 5, I) [9]. These results were also comparable with those for the Hong Kong version (CFI = 0.894) [24] and the Taiwanese version (CFI = 0.886) [25] of the instrument (Table 5, IV).

**Table 5:** Goodness of fit for confirmatory factor analyses of different samples and multiple-group equivalence conditions


	***χ*^2^**	**df**	**RMSEA**	**NNFI**	**CFI**

I. Global Normative (N = 5133)^a^	6830.8	246	0.07	--	0.863
Chronic Illness sample (N = 3313)	3736.9	246	0.07	--	0.876
Nonchronic Illness sample (N = 3862)	4991.3	246	0.07	--	0.868
II. Chinese (N = 1052 without correlated uniqueness)	2401	246	0.101	0.866	0.880
III. Chinese (N = 1052 with correlated uniqueness)	1836	244	0.088	0.898	0.909
Chronic Illness sample (N = 229)	630	244	0.088	0.841	0.860
Nonchronic Illness sample (N = 823)	1471	244	0.089	0.906	0.917
IV. Chinese: Multiple Group analyses (Chronic Illness vs. Nonchronic illness Samples (N = 1052))					
No invariant constraints	2101	488	0.089	0.897	0.909
Loading invariant	2162	512	0.087	0.900	0.907
Loading + factor covariance invariant	2166	518	0.087	0.901	0.907
Loading + factor covariance + uniqueness invariant	2215	544	0.086	0.904	0.905
Mean Structure Model with intercept invariance	2327	564	0.086	0.902	0.900

RMSEA--Root mean square error approximation; NNFI--Non-Normed Fit Index; CFI--Comparative Fit Index;

^a^normative data [9]

To determine whether the factorial structures for the participants with and without chronic illness were similar, multiple group confirmatory analyses were conducted. The results showed that the fit of the model did not decline much when the loadings, factor covariance, and item uniqueness were forced to be invariant between the two groups (Table 5, IV). This suggested that the participants with and without chronic illness shared the same factorial structure, loadings, and reliabilities and thus supported the use of the mainland Chinese version of WHOQOL-BREF for these two populations.

Comparison of the mean scores under the multiple-sample structural equation modelling with mean structure [32] showed that the participants without chronic illness had significantly higher QOL in the physical, psychological, and social relationship dimensions, but not in the environmental dimension; *t *(1052) = 4.156, 2.232, 2.347, -0.684, respectively; all *p *< 0.05 except for the last comparison. This confirmed the results obtained using the means of the items in each scale reported earlier.

### Quality of life outcomes

Table 6 shows the mean and SD of each domain of the WHOQOL-BREF for the total sample in this study as well as the results from the Taiwanese and normative samples [9,27]. Zero-order correlations among the demographic variables were computed (Table 6). To investigate how QOL was related to the socio-demographic variables, four separate multiple stepwise regression analyses were conducted. The score for each QOL dimension was used separately as the criterion variable and the demographic variables were entered as the predictors. Regression analyses, rather than multiple t-tests or analysis of variance, were adopted so that the effects of gender, for example, could be analyzed while the effects of other demographic variables (e.g. age, marital status) were controlled.

**Table 6:** Dimensional scores and regression analyses of demographic variables (standardized beta weights)


	**Physical**	**Psychological**	**Social relationship**	**Environmental**

**Means and Range**				
Guangzhou (N = 1052)				
for scales in the Range 4-20	14.56 ± 2.00	13.69 ± 2.23	14.11 ± 2.23	12.33 ± 2.32
for scales in the Range 0-100	66.00 ± 12.56	60.55 ± 13.96	63.21 ± 13.92	52.04 ± 14.53
Taiwan (N = 13083)^a^	59.12 ± 13.69	49.43 ± 15.63	56.51 ± 14.28	42.38 ± 14.92
Global (23 countries) (N = 11830)^b^	16.20 ± 2.90	15.00 ± 2.8	14.30 ± 3.2	13.50 ± 2.60
**Zero-order correlations with demographic background**				
Sex (Male = 0, Female = 1)	-0.070*	-0.079*	0.014	0.050
Age	-0.250**	-0.095**	-0.094**	0.059
Marital status (unmarried = 0, married = 1)	-0.070*	-0.022	-0.026	0.012
High education (No = 0, Yes = 1)^c^	0.177**	0.185**	0.131**	0.106**
Income	0.111**	0.114**	0.078*	0.102**
Employment status (No = 0, Yes = 1)	-0.196**	-0.111**	-0.101**	-0.034
Chronic illness (No = 0, Yes = 1)	-0.221**	-0.062*	-0.078*	0.018
**Regression analyses with demographic background**^**d**^**(standardized beta)**				
Sex (Male = 0, Female = 1)	-0.022	-0.064*	0.037	0.044
Age	-0.119**	0.007	-0.007	0.149***
Marital status (unmarried = 0, married = 1)	0.037	0.044	0.017	0.000
High education (No = 0, Yes = 1)^c^	0.071*	0.164***	0.114***	0.157***
Income	0.055	0.072*	0.035	0.091**
Employment status (No = 0, Yes = 1)	0.096**	0.051	0.076*	-0.028
Chronic illness (No = 0, Yes = 1)	-0.144***	-0.027	-0.044	-0.007

^a ^Taiwan data [26]; scores in the range 0-100

^b ^Global domain data adjusted for age and sex [9] using scales in the range 4-20

^c ^High education: with a degree, vocational or other training at or above this level

^d ^Standardized beta in regression equations predicting each quality of life dimension

*p < 0.05, **p < 0.01, ***p < 0.001

The results showed that the males had significantly higher QOL in the psychological domain than the females (Table 6). Younger residents had significantly higher physical QOL but significantly lower environmental QOL than older residents. Whereas marital status was not related to QOL, socioeconomic status, measured by levels of education and income, was highly related. Further analyses showed that after controlling for other relevant variables, education had substantially greater positive effects than income. Participants with a higher level of education had significantly higher physical, psychological, social, and environmental QOL than the less educated ones, and wealthier individuals had significantly higher psychological and environmental QOL than poorer ones. Residents without chronic illness had significantly higher physical QOL than those with chronic illness. Furthermore, employed individuals had significantly higher physical and social QOL than those who were unemployed.

### Prevalence of low QOL participants

As discussed in the literature review above, we can set cut-off criteria (standard) for low QOL and estimate the proportion of people with low QOL in the sample (Table 7). Specifically we set (i) "70% of the maximum score" and (ii) "1 SD below the mean" as the cut-off criteria and calculated the proportion of people below these cut-off scores.

**Table 7:** Prevalence of people with low quality of life (all without chronic illness)


**Gender/age (total N)**	**Physical**		**Psychological**		**Social relations**		**Environment**	
	
	**N**	**%**	**N**	**%**	**N**	**%**	**N**	**%**

**Using lower than 70% criterion**								
**Male** + **Female**								
< 44 (589)	298	50.6	400	67.9	373.	67.3	531	90.2
45-59 (143)	89	62.2	102	71.3	98	68.5	128	89.5
> 60 (91)	63	69.2	70	76.9	63	69.2	82	90.1
Total (823)	450	54.7	572	69.5	534	64.9	741	90
**Male**								
< 44 (325)	163	50.2	203	62.5	204	62.8	289	88.9
45-59 (67)	48	71.6	49	73.1	47	70.1	64	95.5
> 60 (63)	49	77.8	52	82.5	51	81	60	95.2
Total (455)	260	57.1	304	66.8	302	66.4	413	90.8
**Female**								
< 44 (342)	190	55.6	249	72.8	222	64.9	313	91.5
45-59 (126)	78	61.9	89	70.6	88	69.8	109	86.5
> 60 (129)	105	81.4	103	79.8	96	74.4	112	86.8
Total (597)	373	62.5	441	73.9	406	68	534	89.4
**Using 1 SD below the mean criterion**								
**Male** + **Female**								
*< 44 (589)*	59	10.0	84	14.3	42	7.1	75	12.7
45-59 (143)	18	12.6	31	21.7	7	4.9	29	20.3
> 60 (91)	20	22.0	16	17.6	8	8.8	8	8.8
Total (823)	97	11.8	131	15.9	57	6.9	112	13.6
**Male**								
< 44 (325)	35	10.8	38	11.7	28	8.6	45	13.8
45-59 (67)	8	11.9	14	20.9	4	6.0	14	20.9
> 60 (63)	18	28.6	11	17.5	6	9.5	10	15.9
Total (455)	61	13.4	63	13.8	38	8.4	69	15.2
**Female**								
< 44 (342)	37	10.8	58	17.0	20	5.8	41	12.0
45-59 (126)	22	17.5	26	20.6	4	3.2	21	16.7
> 60 (129)	34	26.4	18	14.0	6	4.7	7	5.4
Total (597)	93	15.6	102	17.1	30	5.0	69	11.6

The two criteria produced very different figures for the probable prevalence of low QOL in this sample. When the 70% score was used as the cut-off criterion, 54.7-90% of the sample would be considered to have poor QOL, consisting of 57.1-90.8% of men and 62.8-89.4% of women. Using the criterion of < 1 SD, 6.9-15.9% of the subjects had low QOL, consisting of 8.4-15.2% men and 5.0-17% women.

## Discussion

The present study systematically assessed the psychometrical properties of the mainland Chinese version of the WHOQOL-BREF and investigated the differences in QOL between urban residents with and without chronic illness using a moderately large sample. Similar to other instruments, the WHOQOL-BREF has its merits as well as shortcomings, all of which have been highlighted in recent reviews of general measures of QOL [8]. The current findings provide valuable reliability, validity and other useful psychometric information for potential users of the instrument and for researchers who might like to further revise the questionnaire.

The assessment of internal consistency reliability, measured by Cronbach's α, showed that except for the physical domain, all α values were larger than 0.70. Our data showed that Item 3 ('pain and discomfort') and Item 4 ('dependence on medication') were not strongly related to the physical domain, of which the internal consistency would improve substantially had these items been deleted. This is consistent with the previous reports by Skevington et al. [6,8,9], who showed that in 7 out of 24 WHOQOL research centres, the items on pain and dependence on medication showed the lowest correlation with the physical domain. This also indicates that, in the Chinese version as well as in other versions, these items might have meanings that are unique to their respective cultures. The results of reliability and validity further indicate that the two culturally specific items do not add much information to the original 26 items. Future revision of the instrument should address this issue, particularly for the pain and medication items.

In the current study, validity was assessed in three different ways, including item convergent, discriminant, and construct validity. The result showed that all four domains met the criterion for item convergence: each subscale had a significantly higher average item-corrected total correlation for corresponding domains as compared to non-corresponding domains. Discriminant validity was assessed and confirmed by comparing domain scores between community residents with and without chronic illness. The differentiation was the greatest in the physical domain, followed by the social and psychological domains. The lack of differentiation between these two groups for the environmental domain reconfirmed that the environment domain is context rather than health-related. The result is in agreement with the study of Lai et al. [27] and Huang et al. [35] on Chinese people in Taiwan and that of Aigner et al. [39] on patients with chronic illness. This also suggests that the different types of chronic illness endured by the participants generally do not affect the quality of the patients' environmental conditions. Greater effect sizes in QOL between the two contrasting groups may be observed, however, in intervention studies when specific targeted improvements are provided to the patients (e.g., changing the living environment to a nursing home, providing relaxation training) [40]. In summary, the present findings corroborate the previous reports on other Chinese versions of the instrument in a different, but culturally relevant population, and reveal the robust validity of the mainland version to measure the QOL in mainland Chinese people.

The CFA of the WHOQOL-BREF suggested that a four-domain solution was appropriate and that the factorial structure was the same between the participants with and without chronic illness [41]. Specifically, the results showed that the four domains could be clearly differentiated and were potentially equivalent in terms of loadings, factor correlations, and reliabilities among different patient groups. The current findings demonstrate that despite possible culture differences, the mainland Chinese version of WHOQOL-BREF has retained the original factorial structure of the instrument and supported the use of the instrument in diverse populations.

Two cut-off criteria were used to estimate the prevalence of Chinese resident with low QOL [19,20], the 70% maximum score cut-off point criterion produced a prevalence rate of 55-90% low QOL participants which seem to be unreasonably high and harsh. In contrast, the one SD below mean estimates seemed to be more reasonable.

Irrespective of the criterion used, the results of our study suggest that the Chinese in this sample had relatively low QOL in comparison with international data from the Western world [9,42,43]. In line with another Chinese study in Taiwan [26], the domain scores in our study were consistently below those from other countries, particularly in the physical, psychological and environmental domains [9]. This finding of a high prevalence of low QOL population is contradictory to the belief that Chinese have greater endurance, forbearance and tolerance of pain and adverse physical conditions. Whether this is due to the overly rapid economic development in China that might lead to greater dissatisfaction and negative feelings has yet to be investigated.

Consistent with previous research [44,45], socioeconomic status was positively related to QOL in the Chinese urban community residents. Among various sociodemographic variables, the level of education had the strongest relationships with the four domains of QOL. More importantly, the results of our further analyses showed that education had a much stronger effect on QOL than income, suggesting that education plays a greater role in maintaining health and higher QOL among mainland Chinese than wealth. This finding could stimulate further discussion and probably research in both academics circles and the general public in the Chinese mainland.

Age was associated with the physical and environmental domains of QOL. Older community residents had worse physical but better environmental QOL than younger residents, which could be explained by their worsened health status but relatively adequate provision of housing before retirement for their civil or semi-government services. Gender differences, after controlling for other demographic factors, were relatively small. The effect of gender was statistical significant only for the psychological domain of QOL. In congruence with other studies [42,43], male participants were found to have better psychological QOL than females, possibly because males have a competitive advantage over females in work and social situations. This may also reflect deeply a rooted male dominance culture which exists in many Asian societies, including China.

The main limitations of this study are: (i) as the sample was drawn from only one city, the population surveyed is not demographically diverse enough to be representative of all Chinese in the mainland and thus generalization of the results must be considered carefully; (ii) the high rate of missing data for Item 21 ('sexual life'). The latter issue was consistent with previous Chinese [45] or Western studies [9] (e.g., 6% in Skevington [9]). This could indicate the suppressed expression of sexual desire and the unwillingness to talk to strangers about such a sensitive issue. Additional studies, with more carefully designed questions to elicit people's frank responses on such issues, are in great need and will help better elucidate the real role that sexual life is playing in the overall QOL. Other than this item on sexual life, the low missing rate (less than 0.3%) was in line with previous large scale international studies [less than 1% in Skevington [9]].

## Conclusions

The mainland Chinese version of the WHOQOL-BREF questionnaire is a convenient and useful instrument for assessing the QOL in a large-scale Chinese population. Subjective QOL was found to be low among our sample of Chinese urban community residents and was influenced by socio-demographic variables. Specifically, education was found to be the most important factor that affected the QOL of our sample of Chinese people. This study has provided preliminary psychometric information on the reliability and validity of the WHOQOL-BREF as used in mainland China, which helps researchers' interpretation of findings in relevant studies as well as their future revision of the instrument.

## Competing interests

The authors declare that they have no competing interests.

## Authors' contributions

PX conceived and coordinated the whole project and analyzed the data. PX and KTH wrote the manuscript. NL and CL helped in data analyses and revision of various drafts. YL coordinated the field work. All authors read and approved the final manuscript.

## Pre-publication history

The pre-publication history for this paper can be accessed here:

http://www.biomedcentral.com/1471-2288/12/37/prepub
